# Factors associated with wait times across the breast cancer treatment pathway in Ontario

**DOI:** 10.1186/2193-1801-2-388

**Published:** 2013-08-19

**Authors:** Amalia Plotogea, Anna M Chiarelli, Lucia Mirea, Maegan V Prummel, Nelson Chong, Rene S Shumak, Frances P O’Malley, Claire M B Holloway, the Breast Screening Study Group

**Affiliations:** Prevention and Cancer Control, Cancer Care Ontario, 620 University Avenue, Toronto, ON M5G 2L7 Canada; Dalla Lana School of Public Health, University of Toronto, Toronto, Canada; Maternal-Infant Care Research Centre, Mount Sinai Hospital, Toronto, Canada; Institute for Clinical Evaluative Sciences, Toronto, Canada; Department of Laboratory Medicine and Pathobiology, University of Toronto and St. Michaels Hospital, Toronto, Canada; Women’s College Hospital Sunnybrook Health Sciences Centre, Toronto, Canada

**Keywords:** Breast cancer, Treatment wait time, Surgery, Radiotherapy, Chemotherapy, Demographic factors

## Abstract

**Background:**

Longer times from diagnosis to breast cancer treatment are associated with poorer prognosis. This study examined factors associated with wait times by phase in the breast cancer treatment pathway.

**Methods:**

There were 1760 women eligible for the study, aged 50–69 diagnosed in Ontario with invasive breast cancer from 1995–2003. Multivariate logistic regression examined factors associated with greater than median wait times for each phase of the treatment pathway; from diagnosis to definitive surgery; from final surgery to radiotherapy without chemotherapy and from final surgery to chemotherapy.

**Results:**

The median wait times were 17 days (Inter Quartile Range (IQR) = 0–31) from diagnosis to definitive surgery, 44 days (IQR = 34–56) from final surgery to postoperative chemotherapy and 75 days (IQR = 57–97) from final surgery to postoperative radiotherapy. Diagnosis during 2000–2003 compared to 1995–1999 was associated with significantly longer wait times for each phase of the treatment pathway. Higher income quintile was associated with longer wait time from diagnosis to surgery (OR = 1.47, 95% CI = 1.05-2.06) and shorter wait times from final surgery to radiotherapy (OR = 0.60, 95% CI = 0.37-0.96). Greater stage at diagnosis was associated with shorter wait times from diagnosis to definitive surgery (stage III vs I: OR = 0.49, 95% CI = 0.34-0.71).

**Conclusions:**

While diagnosis during the latter part of the study period was associated with significantly longer wait times for all phases of the treatment pathway, there were variations in the associations of stage and income quintile with wait times by treatment phase. Continued assessment of factors associated with wait times across the breast cancer treatment pathway is important, as they indicate areas to be targeted for quality improvement with the ultimate goal of improving prognosis.

## Introduction

The improved survival for women diagnosed with breast cancer in Canada has been attributed to a combination of early detection through mammography screening and advances in evidence based treatment protocols (Canadian Cancer Society’s Steering Committee 2011). Treatment for breast cancer is multifaceted, and both the use of loco-regional radiotherapy (Whelan et al. 2000) and systemic chemotherapy (Baum 1998) has been shown to improve breast cancer survival over use of surgery alone. However, despite the improved survival outcomes, wait times across the entire patient diagnosis and treatment pathways remain a concern. A review examining symptomatic cancers found that a 3–6 month delay from onset of symptoms to first treatment was associated with overall shorter survival among breast cancer patients (Richards et al. 1999). Reviews examining treatment specific outcomes have found that wait times to postoperative chemotherapy of greater than 4 weeks are associated with shorter disease-free and overall survival (Balduzzi et al. 2010), while wait times longer than 8 weeks to initiation of postoperative radiotherapy have been associated with higher local recurrence rates, but without impact on survival (Chen et al. 2008; Huang et al. 2003). A large Canadian study examining women diagnosed with early stage breast cancer did find higher local and distant recurrence rates and inferior breast cancer survival among women who began radiotherapy more than 20 weeks following surgery, compared to those who began within 4 to 8 weeks (Olivotto et al. 2009).

In Canada, later stage at diagnosis (Rayson et al. 2004) and receipt of radiotherapy compared to chemotherapy (Rayson et al. 2007) has been associated with longer wait time to postoperative treatment in symptomatic women. Additionally, being diagnosed greater than 70 years of age has been associated with greater wait time from diagnosis to first treatment (Rayson et al. 2004; Olivotto et al. 2001). A study from Manitoba, Canada found a periodic association between rural residence and shorter treatment intervals, specifically among women diagnosed in early 2001 and not in a more recent cohort, diagnosed in 2005 (Cooke et al. 2009). The majority of these studies included symptomatic women only.

Factors associated with variation in treatment wait times may provide an opportunity for quality improvement with the ultimate goal of improving prognosis for women diagnosed with breast cancer. The present cohort included Ontario women diagnosed between 1995 and 2003 with screen-detected, interval, or symptomatic breast cancers. The main objective of this study is to examine demographic, prognostic and clinical factors associated with wait times across defined phases of the treatment pathway; namely, from invasive breast cancer diagnosis to definitive surgery, from final surgery to radiotherapy when chemotherapy is not given, and from final surgery to chemotherapy prior to radiotherapy.

## Methods

Ethics approval for this study was granted by the University of Toronto Health Sciences Research Ethics Board, the Regional Cancer Centers and Princess Margaret Hospital. Methods were described thoroughly in a previous study (Chiarelli et al. 2012). Briefly, a cohort of women (N = 807,966) between the ages of 50–63 as of Jan 1, 1995 who registered for health care benefits through the Ontario Health Insurance Plan (OHIP) was identified. The cohort was linked to women in the Ontario Cancer Registry (OCR) to ascertain the date of diagnosis of primary invasive breast cancer, of any histological type, from Jan 1, 1995 to Dec 31, 2003. Women identified with prior history of breast cancer (n = 15,684), unknown sex (n = 3), who were not residents of Ontario (n = 7,633), less than 50 years of age (n = 33) or who had died before the start of the study (n = 12,898) were excluded. Information on mammograms performed through OHIP was obtained by merging cohort data with OHIP files, and extracting all physician claims for bilateral mammography during the study period, with an algorithm to distinguish between screening and diagnostic mammograms (Chiarelli et al. 2012). Information from women screened within the Ontario Breast Screening Program (OBSP) was obtained from data routinely collected by an integrated client management system. Since 1990, the OBSP has offered eligible women biennial screening consisting of two view mammography. A complete description of the details of OBSP has been published (Chiarelli et al. 2006). All identified OBSP and OHIP screens were merged to a screening history for each woman.

**Figure 1:**
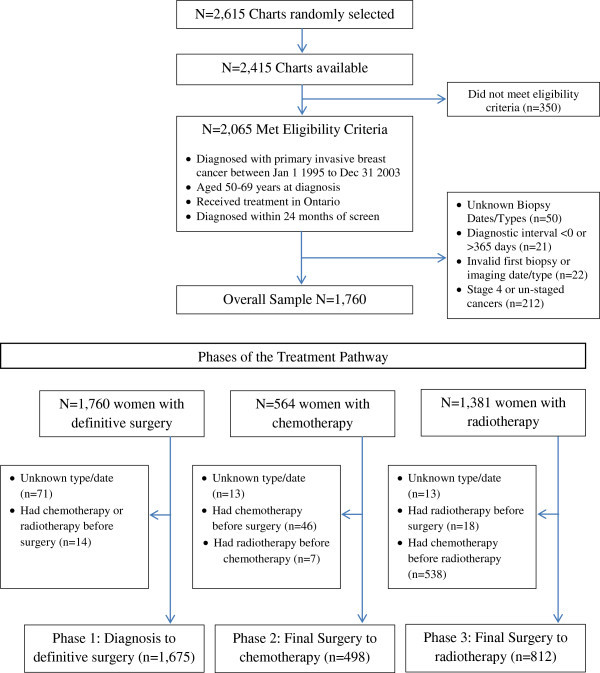
**Flow chart of study population leading to final cohorts for treatment analysis.**

Of the 16,373 invasive breast cancer cases that occurred in Ontario between 1995 and 2003, 2,615 were randomly selected for chart abstraction. Of the 2,415 women with available charts, 350 did not meet the eligibility criteria (specified in Figure 1). A woman’s breast cancer was classified as screen-detected if she had a mammogram during the study period, either through OHIP or OBSP, and her breast cancer was diagnosed within 6 months of that screen. Interval breast cancers were defined as cancers that occurred within 6 to12 months of a mammogram. Symptomatic breast cancers were cancers diagnosed in women who did not have a screening mammogram during the study period prior to diagnosis.

### Phases in the treatment pathway

Wait times were examined across phases in the treatment pathway from invasive breast cancer diagnosis to definitive surgery; from final surgery to initiation of radiotherapy without chemotherapy and from final surgery to chemotherapy prior to radiation. Definitive surgeries included those occurring within 4 months of a tissue diagnosis. A definitive surgery was defined for each woman as the first procedure after diagnosis, coded as partial mastectomy (with a prior FNA or core biopsy), mastectomy, axillary lymph node dissection (ALND) or Sentinel Lymph Node Biopsy (SLNB). Wait time to postoperative adjuvant treatment was calculated from the date of final surgery following diagnosis (which included incisional/excisional biopsy, mastectomy, ALND, SLNB and re-excisions) to the date of first adjuvant chemotherapy or breast radiotherapy. Adjuvant treatment included those occurring within 1 year of diagnosis.

### Definition of covariates

Information on tumour characteristics was abstracted from pathology and surgical reports included in the medical charts (Chiarelli et al. 2012). The TNM classification scheme was used for staging of breast cancer (American Joint Committee on Cancer 2002). Tumour size was defined as the largest diameter of the invasive carcinoma. Among women who had axillary assessment with either sentinel lymph node biopsy or axillary node dissection, lymph node status was defined as positive by TNM criteria. Biopsies included those conducted within a day of the diagnosis date and were either percutaneous, (FNA and core biopsies) or surgical (excisional biopsy or partial mastectomy). OCR data was used to obtain age and date at diagnosis and treatment center location. Treatment center was classified as the cancer center the woman first attended, and grouped according to geographic region. South Central region included Toronto and Hamilton, South Eastern region included Ottawa and Kingston, South Western region included London and Windsor and Northern region included Sudbury and Thunder Bay. Postal codes from either residence at first screen (interval and screen-detected cases) or the start of the study period, January 1, 1995 (symptomatic cases) were linked to the 2001 Canadian census to obtain neighborhood income quintile (Chiarelli et al. 2012).

### Statistical analysis

Clinical characteristics and treatments received were examined overall and by detection method. For each phase in the treatment pathway, the distribution of wait times was quantified by the median and interquartile range (IQR). As treatment wait times were skewed, with more women experiencing relatively shorter compared to longer wait times, comparisons of wait times between detection groups were performed using the non-parametric Wilcoxon Rank sum test. Logistic regression analysis examined associations between factors and treatment wait times, dichotomized as greater or less than the observed median. Adjusted odds ratios (OR) and 95% confidence intervals (95%CI) were estimated to quantify associations, and statistical significance was evaluated using 2-sided p-values at the 5% testing level. All statistical analyses were performed using SAS 9.2 (SAS Institute Inc 2008).

## Results

Of 2065 eligible women diagnosed with invasive breast cancer, 1760 had valid biopsy types and dates, and were diagnosed at stages I to III (Figure 1). There were 1675 women included in the diagnosis to definitive surgery wait time analysis, 498 women included in the surgery to chemotherapy analysis and 812 women in the surgery to radiotherapy analysis, including women receiving endocrine therapy (see Figure 1 for exclusions). There were a greater number of interval cancers (p = 0.02), and fewer symptomatic cancers (p = 0.01), compared to screen-detected cancers diagnosed in the latter time period (Table 1). Both symptomatic and interval cancers were more likely to be diagnosed at stage III vs. I (p < 0.001), and be treated with chemotherapy (p < 0.01) compared to screen-detected cancers. Symptomatic cancers were also significantly less likely to have radiotherapy compared to screen-detected cancers, although a greater proportion also had mastectomy.

**Table 1:** **Distribution of clinical characteristics and treatment received overall, and among women diagnosed with screen-detected, interval and symptomatic breast cancers (n = 1,760)**

	Overall	Screen-detected	Interval	Symptomatic
	N = 1,760	N = 1096	N = 309	N = 355
Characteristics	N (%)	N (%)	N (%)	N (%)
**Age Category**				
50-59	680 (38.6)	415 (37.9)	124 (40.1)	141 (39.7)
60-69	1080 (61.4)	681(62.1)	185 (59.9)	214 (60.3)
**Diagnosis Year** ^*^				
1995-1999	974 (55.3)	602 (54.9)	147 (47.6)	225 (63.4)
2000-2003	786 (44.7)	494 (45.1)	162 (52.4)	130 (36.6)
**Chemotherapy** ^†^				
No chemotherapy	1098 (62.4)	728 (66.4)	177 (57.3)	193 (62.8)
Chemotherapy	662 (37.6)	368(33.6)	132 (42.7)	162 (37.2)
**Radiotherapy** ^**a**‡^				
No Radiotherapy	370 (21.2)	218 (19.9)	58 (18.8)	94 (26.5)
Radiotherapy	1377 (78.8)	873 (79.7)	246 (79.6)	258 (72.7)
**Definite Surgery** ^**b**δ^				
Incisional/excisional	112 (6.6)	79 (7.5)	14 (4.7)	19 (5.8)
Partial Mastectomy	1071 (63.4)	688 (64.8)	185 (62.0)	198 (60.0)
Total Mastectomy	101 (6.0)	55 (5.2)	24 (8.1)	22 (6.7)
Modified/Radical Mast.	328 (19.4)	185 (17.4)	59 (19.8)	84 (25.5)
ALND/SLNB	77 (4.6)	54 (5.1)	16 (5.4)	7 (2.1)
**TNM Stage** ^**^				
1	925 (52.6)	662 (60.4)	161 (52.1)	102 (28.7)
2	603 (34.3)	332 (30.3)	97 (31.4)	174 (49.0)
3	232 (13.2)	102 (9.3)	51 (16.5)	79 (22.3)

^a^Unknown radiotherapy n = 13.

^b^unknown surgery type n = 27; invalid surgery type n = 44.

*difference for interval (p = 0.02) and symptomatic (p = 0.01) compared to screen-detected.

^†^difference for interval (p = 0.003) and symptomatic (p < 0.001) compared to screen-detected.

^‡^difference for symptomatic compared to screen-detected (p = 0.01).

^δ^difference for symptomatic compared to screen-detected (p = 0.003).

**difference for interval (p < 0.001) and symptomatic (p < 0.001) compared to screen-detected.

Overall, the most common treatments were surgery with radiotherapy (47.6%) and a combination of surgery, chemotherapy and radiotherapy (31.4%) (Table 2). The percentage of women treated with surgery and radiotherapy was 52%, 45% and 36% for women with screen-detected, interval and symptomatic cancers, respectively. An opposite pattern was observed for treatment with surgery, chemotherapy and radiotherapy where 29%, 36% and 37% of women were screen-detected, interval and symptomatic, respectively (Table 2). The median wait time from diagnosis to definitive surgery was 17 days (IQR = 0-31 days), with 10% of women waiting 49 days or more. The median wait times from surgery to chemotherapy was 44 days (IQR = 34-56 days), with 10% of women waiting longer than 70 days. Among women who did not receive chemotherapy, the median wait time to radiotherapy was 75 days (IQR = 57-97 days), with 10% of women waiting 118 days or longer. There were no significant differences in median wait times within any phase of the treatment pathway between screen-detected, interval or symptomatic cancers.

**Table 2:** **Description of wait times to definitive surgery, radiotherapy and chemotherapy overall, and among women diagnosed with screened, interval and symptomatic breast cancers**

	Overall	Screen-detected	Interval	Symptomatic
	N = 1760	N = 1096	N = 309	N = 355
	N (%)	N (%)	N (%)	N (%)
**Treatments Received** ^a^				
Surgery + Chemotherapy	110 (6.3)	60 (5.5)	20 (6.5)	30 (8.5)
Surgery + Radiotherapy	838 (47.6)	570 (52.0)	139 (45.0)	129 (36.2)
Surgery + Chemotherapy + Radiotherapy	552 (31.4)	308 (29.0)	112 (36.2)	132 (37.2)
Surgery Only	260 (14.8)	158 (14.4)	38 (12.3)	64 (18.0)
**Diagnosis to Definitive Surgery** ^**b**^				
Number In analysis	N = 1675	N = 1056	N = 293	N = 326
Median Wait Time to Definitive Surgery	17 days	18 days	20 days	15 days
IQR	0-31 days	0-31 days	0-33 days	0-30 days
90th Percentile >	49 days	49 days	51 days	49 days
**Final Surgery to Chemotherapy** ^**c**^				
Number in analysis	N = 498	N = 290	N = 98	N = 110
Median Wait Time to Chemotherapy	44 days	43 days	43 days	46 days
IQR	34-56 days	33-56 days	35-58 days	34-54 days
90th Percentile >	70 days	71 days	71 days	66 days
**Final Surgery to Radiotherapy** ^**d**^				
Number In analysis	N = 812	N = 556	n = 132	N = 124
Median Wait Time to Radiotherapy	75 days	75 days	73.5 days	78 days
IQR	57-97 days	57-96 days	59-97 days	59-100 days
90th Percentile >	118 days	118 days	112 days	138 days

^a^Pearson chi-squared test; difference in treatments received for interval (p = 0.031) and symptomatic (p < 0.001) compared to screen-detected.

^b^Wilcoxon rank sum test; difference in wait times for interval (p = 0.137) and symptomatic (p = 0.191) compared to screen-detected.

^c^Wilcoxon rank sum test; difference in wait times for interval (p = 0.504) and symptomatic (p = 0.573) compared to screen-detected.

^d^Wilcoxon rank sum test; difference in wait times for interval (p = 911) and symptomatic (p = 0.170) compared to screen-detected.

After controlling for demographic and prognostic factors, diagnosis during 2000–2003 compared to 1995–1999 was associated with significantly greater wait time from diagnosis to definitive surgery (OR = 2.29, 95% CI = 1.85-2.86), from final surgery to chemotherapy (OR = 1.62, 95%CI = 1.10-2.41) and from final surgery to radiotherapy (OR = 2.55, 95%CI 1.86-3.52) (Table 3). Compared to those who had treatment in the South Central region, having treatment in the South Eastern region of Ontario was associated with longer wait times to definitive surgery (OR = 1.96, 95%CI = 1.51-2.56) and to chemotherapy (OR = 1.76, 95% CI = 1.12-2.78), but shorter wait time to radiotherapy (OR = 0.40, 95%CI = 0.27-0.58). Having treatment in the South Western region was associated only with an increased wait time to definitive surgery (OR = 1.36, 95%CI = 1.03-1.81), while having treatment in the Northern region was associated with shorter wait times to definitive surgery (OR = 0.57, 95%CI = 0.41-0.81) and to radiotherapy (OR = 0.33, 95%CI = 0.20-0.55). Higher income quintile was associated with greater wait times from diagnosis to definitive surgery (OR = 1.49, 95%CI = 1.05-2.09 for Q2 vs. Q1; OR = 1.47, 95%CI = 1.05-2.06 for Q4 vs. Q1) and shorter wait times from surgery to radiotherapy (OR = 0.60, 95%CI = 0.37-0.96 for Q5 vs. Q1). Women diagnosed with stage III compared to stage I breast cancers were less likely to experience greater wait time to definitive surgery (OR = 0.49, 95%CI = 0.34-0.71) while no differences were observed for the wait times to other treatments.

**Table 3:** **Adjusted odds ratios (OR) and 95%CI describing factors associated with wait times from diagnosis to definitive surgery (n = 1,675), and from final surgery to postoperative chemotherapy (n = 498) or postoperative radiotherapy (n = 812)**

	Diagnosis → Definitive surgery^a^(n = 1,675)			Surgery to chemotherapy^b^(n = 498)			Surgery to radiotherapy^c^( n = 812)		
	Wait time ≤17 days n (%)	Wait time >17 days n (%)	Odds ratio (95% CI)	Wait time ≤44 days n (%)	Wait time >44 days n (%)	Odds ratio (95% CI)	Wait time < 75 days n (%)	Wait time >75 days n (%)	Odds ratio (95% CI)
**Detection Method**									
Screened	527 (62.6)	529 (63.5)	1.00 reference	155 (60.3)	135 (56.0)	1.00 reference	281 (68.9)	275 (68.1)	1.00 reference
Interval	133 (15.8)	160 (19.2)	1.14 (0.86-1.51)	50 (19.5)	48 (19.9)	1.01 (0.62-1.64)	68 (16.7)	64 (15.8)	0.92 (0.60-1.38)
Symptomatic	182 (21.6)	144 (17.3)	0.93 (0.71-1.24)	52 (20.2)	58 (24.1)	1.35 (0.84-2.19)	59 (14.5)	65 (16.1)	1.09 (0.70-1.68)
**Age at Diagnosis**									
50-59	364 (43.2)	285 (34.2)	1.00 reference	129 (50.2)	97 (40.3)	1.00 reference	155 (38.0)	125 (30.9)	1.00 reference
60-69	478 (56.8)	548 (65.8)	0.97 (0.67-1.40)	128 (49.8)	144 (59.8)	1.45 (0.98 -2.14)	253 (62.0)	279 (69.1)	1.12 (0.82-1.55)
**Diagnosis Year**									
1995-1999	555 (65.9)	374 (44.9)	1.00 reference	141 (54.9)	100 (41.5)	1.00 reference	281 (68.9)	197 (48.8)	1.00 reference
2000-2003	287 (34.1)	459 (55.1)	2.29 (1.85-2.86)*	116 (45.1)	141 (58.5)	1.62 (1.10-2.41)^‡^	127 (31.1)	207 (51.2)	2.55 (1.86-3.52)*
**Income Quintile**									
Q1 (poorest)	167 (20.0)	130 (15.7)	1.00 reference	45 (17.7)	44 (18.5)	1.00 reference	63 (15.6)	72 (18.0)	1.00 reference
Q2	140 (16.8)	165 (20.0)	1.49 (1.05-2.09)^‡^	40 (15.7)	48 (20.2)	1.26 (0.68-2.34)	78 (19.3)	65 (16.2)	0.68(0.41-1.12)
Q3	162 (19.4)	155 (18.8)	1.21 (0.86-1.71)	52 (20.4)	42 (17.7)	0.78 (0.42-1.45)	68 (16.8)	85 (21.2)	0.90 (0.54-1.48)
Q4	154 (18.4)	178 (21.6)	1.47 (1.05-2.06)^‡^	54 (21.2)	48 (20.2)	0.96 (0.53-1.75)	77 (19.0)	87 (21.7)	0.84 (0.51-1.38)
Q5 (richest)	212 (25.4)	198 (24.0)	1.16 (0.84-1.61)	64 (25.1)	56 (23.5)	0.84 (0.47-1.50)	119 (29.4)	92 (22.9)	0.60 (0.37-0.96)^†^
**Treatment Centre Region**									
South Central	423 (50.2)	332 (39.9)	1.00 reference	123 (47.9)	99 (41.1)	1.00 reference	156 (38.2)	199 (49.3)	1.00 reference
South Eastern	155 (18.4)	253 (30.4)	1.96 (1.51-2.56)*	60 (23.4)	76 (31.5)	1.76 (1.12-2.78)^∂^	129 (31.6)	68 (16.8)	0.40 (0.27-0.58)*
South Western	137 (16.3)	173 (20.8)	1.36 (1.03-1.81)^†^	31 (12.1)	41 (17.0)	1.67 (0.94-2.94)	58 (14.2)	102 (25.3)	1.24 (0.83-1.87)
Northern	127 (15.1)	75 (9.0)	0.57 (0.41-0.81)*	43 (16.7)	25 (10.4)	0.59 (0.32-1.09)	65 (15.9)	35 (8.7)	0.33 (0.20-0.55)*
**TNM Stage**									
1	432 (51.3)	460 (55.2)	1.00 reference	57 (22.2)	46 (19.1)	1.00 reference	323 (79.2)	312 (77.2)	1.00 reference
2	296 (35.2)	284 (34.1)	0.86 (0.68-1.08)	138 (53.7)	132 (54.8)	1.15 (0.69-1.88)	80 (19.6)	83 (20.5)	1.18 (0.80-1.74)
3	114 (13.5)	89 (10.7)	0.49 (0.34-0.71)*	62 (24.1)	63 (26.1)	1.19 (0.66-2.13)	5 (1.2)	9 (2.2)	2.83 (0.74-10.8)

^a^Adjusted for: detection method, age category, diagnosis year, treatment centre, income quintile, stage and definitive surgery type.

^b^Adjusted for: detection method age category, diagnosis year, treatment centre, income quintile, stage and last surgery type and hormone status.

^c^Adjusted for: detection method, age category, diagnosis year, treatment centre, income quintile, stage and last surgery type.

*p < 0.001; ^†^p = 0.05; ^‡^p = 0.02; ^∂^p = 0.01.

## Discussion

In this cohort of Ontario women, over 75% had wait times within current clinical practice guidelines from diagnosis to definitive surgery or final surgery to postoperative chemotherapy and about 50% had acceptable wait times to postoperative radiotherapy without chemotherapy. Regardless of phase in the treatment pathway, wait times did not differ significantly by detection method. Factors associated with wait times differed by phase of the treatment pathway, except for time period where wait times were consistently significantly longer for women diagnosed in the latter period of study compared to earlier for all phases. Higher stage at diagnosis was significantly associated with shorter wait times to definitive surgery, but not with wait times to any post-surgical treatment. Higher income quintile was also significantly associated with longer wait time to surgery, and shorter times to radiotherapy. In addition, there were substantial regional differences in wait times during this period.

The most common breast cancer treatment for women with screen-detected or interval cancer was breast surgery with radiotherapy. This is expected as breast irradiation following breast conserving surgery was a standard treatment in Canada during the study period (Whelan et al. 2003). Compared to screen-detected cancers, a greater proportion of symptomatic cancers received mastectomy, which may explain the significantly lower odds of post-surgical radiotherapy in this group. Whether a woman had screen-detected, compared to interval or symptomatic breast cancer, was not a major predictor of wait times from diagnosis to definitive surgery, or to post-surgical chemotherapy or radiotherapy. This finding suggests that once a woman is diagnosed with cancer, the detection method does not substantially impact wait times to treatments.

The Canadian Steering Committee on Clinical Practice Guidelines for the Care and Treatment of Breast Cancer currently recommends that local breast irradiation not accompanied by chemotherapy should commence within 12 weeks after surgery (Whelan et al. 2003) and that adjuvant chemotherapy begin as soon as possible following surgical healing (Cancer Care Ontario 2010). In our cohort, the median times to postoperative chemotherapy (6.3 weeks) and radiotherapy (10 weeks), were within these clinically acceptable time frames. However, the upper 10% of women experienced intervals of greater than 10.0 weeks for chemotherapy. It has been shown that relapse free survival and overall survival are impacted when a women begins chemotherapy more than 10 to 12 weeks following surgery (Shannon et al. 2003; Baum 1998; Balduzzi et al. 2010). Among women who received postoperative radiotherapy, 10% had treatment intervals of greater than 17 weeks. A recent population based Canadian study examining impact of treatment times among women diagnosed with early stage breast cancer, found increased recurrence rates and inferior breast cancer specific survival for women who began radiotherapy greater than 20 weeks following surgery, compared to within 4–8 weeks (Olivotto et al. 2009). We found that 90% of women received surgery within 7 weeks of their diagnosis, with median time to surgery of 2.4 weeks, which was lower than 12 weeks, the recommendation during the study period (Canadian Breast Cancer Network 2008).

Being diagnosed with breast cancer in the latter part of the study, 2000 to 2003, compared to the earlier, 1995 to 1999, was associated with an increased wait time from diagnosis to definitive surgery and also with wait time from surgery to both chemotherapy and radiotherapy. Similarly a study conducted in Nova Scotia, Canada during a similar time period also found that time to first adjuvant therapy (chemotherapy or radiotherapy), following surgery was substantially longer for the more recent cohort(2003/2004) compared to the more distant one(1999/2000) (Rayson et al. 2007). Longer wait times for radiotherapy in the later cohort may be explained by a shortage of radiation therapists, medical physicists and radiation oncologists in the late 1990’s, such that some patients had to be seen out of province for treatment (D’Souza et al. 2001; Randal 2000). The explanations for lengthening wait times for definitive surgery and chemotherapy are less readily apparent, but may include increasing cancer incidence with disproportionately smaller increases in resources for cancer surgery and increased use of chemotherapy for node negative breast cancer in the later time period.

Regional differences in wait times were observed in this cohort. Compared to having treatment in the South-Central region of the province, having treatment in the North was associated with shorter wait times to radiotherapy and having treatment in the South East region was associated with longer wait times to definitive surgery, and chemotherapy, but shorter wait time to radiotherapy. Similar regional variation in radiotherapy wait times have been observed in Ontario among women not receiving chemotherapy (Olivotto et al. 2009). This may also be explained by the shortage of radiation therapists, physicists and radiation oncologists in different regions (D’Souza et al. 2001; Randal 2000). A variety of systemic, organizational and procedural differences, including capacity to provide therapy, may be concurrently contributing to the observed variation (Benk et al. 2006). Regional variation in wait times continue to be monitored as an important indicator of the cancer delivery system across the province (Cancer Care Ontario 2010).

Contrary to other literature, we did not find age at diagnosis to be associated with wait times for any of the treatment phases. While other studies included women over the age of 70, ours included women up to age 69 only, which may explain some of the discrepancies (Rayson et al. 2004; Caplan et al. 2000). We found that increasing income quintile was associated with shorter wait time to radiotherapy, but with longer time to definitive surgery. Our study is not directly comparable to other studies which have assessed individual income as a predictor of wait time to treatment, as we have used an area level income measure categorized into quintiles. A study out of Montreal, Canada, which examined personal income as a predictor of wait times among women diagnosed with early stage breast cancer did not find any association between median income and delay in post-surgical radiotherapy (classified as more than 7 weeks without chemotherapy, and more than 24 weeks with chemotherapy) (Benk et al. 1998). Wait time from diagnosis to definitive surgery was substantially shorter for women diagnosed with stage III compared to stage I cancers. This observation is consistent with the expedition of diagnosis and treatment of more severe cases. Expedition of treatment for cancers at higher stages has been seen in other Canadian provinces (Caplan et al. 2000), as has expedition to diagnosis of more suspect cases (Olivotto et al. 2001; Arndt et al. 2003). Alternatively, women diagnosed at a higher stage were less likely to receive breast conserving surgery; the decision to conserve the breast may necessitate other investigations post diagnosis but before surgery to accurately define the volume of breast tissue to be removed. Such investigations often contribute to longer wait times from diagnosis to surgery. Notably, stage did not affect post-surgical adjuvant treatment wait times in our study.

Strengths of this study include the large cohort of breast cancer cases identified through the OCR. Evaluation of the OCR suggests a high level of completeness and accuracy for breast cancer ascertainment (Holowaty et al. 1995). Furthermore, through the chart abstraction, we were able to obtain detailed information on type and dates of treatment procedure and staging of the diagnosed breast cancer cases. Several limitations should be addressed. Currently, Ontario measures wait times from ready to treat dates, which we did not collect, thus our findings are not directly comparable to current standards. Additionally, although wait times are sometimes seen as a reflection of the cancer system efficiency, these wait time intervals may be influenced by a variety of patient and system level factors, such as delays in seeking treatment, scheduling appointments or availability of specialists, which are not captured by our data. Also, it is important to note that this is a historic cohort, and continued assessment is required to evaluate factors associated with wait times as breast cancer treatment protocols and practices evolve.

In our study, over 75% of Ontario women diagnosed with breast cancer from 1995 to 2003 had wait times from diagnosis to definitive surgery or final surgery to postoperative chemotherapy within current clinical practice guidelines; whereas, about half of the women had acceptable wait times from final surgery to postoperative radiotherapy without chemotherapy. Breast cancer detection method did not substantially impact wait times by treatment pathway. While stage of cancer was a determinant of wait times to definitive surgery, it was not associated with time to post-surgical chemotherapy or radiotherapy. Other factors associated with longer wait times that varied by phase of the treatment pathway included income quintile and region of cancer treatment centre. More recent time period of diagnosis (2000–2003) was associated with longer wait times for all phases of the treatment pathway. Continued assessment of factors associated with wait times from diagnosis to surgery and to post-surgical treatment is important, as they indicate areas to be targeted for quality improvement with the ultimate goal of improving prognosis for women diagnosed with breast cancer.

## Author’s information

The Breast Screening Study Group

Members include: Norman Boyd, Campbell Family Institute for Breast Cancer Research, Ontario Cancer Institute; Lynn Chappell, Erie St Clair Regional Cancer Program; Brenda Fleming, London Regional Cancer Program; Amanda Hey, Health Sciences North; Suzie Joanisse, Ottawa Hospital Cancer Centre; Alison McMullen, Regional Cancer Care Northwest; Carol Rand, Juravinski Cancer Centre; Lori Van Manen, Southeast Regional Cancer Program.
